# Analysis of radioactivity in commercially available products aiming to improve health and wellness

**DOI:** 10.1093/rpd/ncad192

**Published:** 2023-07-05

**Authors:** Rimon Thomas, Eva Forssell-Aronsson, Martin Hjellström, Klara Insulander Björk, Francisco Piñero-García, Mats Isaksson

**Affiliations:** Department of Medical Radiation Sciences, Institute of Clinical Sciences, Sahlgrenska Academy at University of Gothenburg, Gothenburg SE-413 45, Sweden; Department of Medical Radiation Sciences, Institute of Clinical Sciences, Sahlgrenska Academy at University of Gothenburg, Gothenburg SE-413 45, Sweden; Department of Medical Physics and Biomedical Engineering, Sahlgrenska University Hospital, Gothenburg SE-413 45, Sweden; Department of Medical Radiation Sciences, Institute of Clinical Sciences, Sahlgrenska Academy at University of Gothenburg, Gothenburg SE-413 45, Sweden; Department of Medical Radiation Sciences, Institute of Clinical Sciences, Sahlgrenska Academy at University of Gothenburg, Gothenburg SE-413 45, Sweden; Department of Medical Radiation Sciences, Institute of Clinical Sciences, Sahlgrenska Academy at University of Gothenburg, Gothenburg SE-413 45, Sweden; Department of Medical Radiation Sciences, Institute of Clinical Sciences, Sahlgrenska Academy at University of Gothenburg, Gothenburg SE-413 45, Sweden

## Abstract

There are products available on the online market that are claim to contain unique ‘energies’ that can improve health and wellness by eliminating toxins and pains and energising food and drinking water. We investigated these products by alpha and gamma spectrometry, and the analysis showed that they contained a few to hundreds of kilobecquerels per kilogram of naturally occurring radionuclides from the ^232^Th and ^238^U series. The committed effective dose for an adult drinking water that had been in contact with these products just once was estimated to 12 nSv. Considering a worst-case scenario for the workers inhaling the radioactive substance, 1 d of work would result in an effective dose of 0.39 mSv. The product descriptions do not mention the radionuclide content, and concerns are raised for the consumers and workers exposed to these products with no knowledge of the radioactive content.

## Introduction

Since the discovery of uranium by Martin Klaproth in 1789, and its radiation (by an accident) by Henri Becquerel in 1896, radionuclides and radioactivity have been an integral part of human society such as in the areas of medicine, military and various industries. After the displacement law of radioactivity was discovered in 1913 by Frederick Soddy and Ernest Rutherford, the decay series of ^238^U and ^232^Th could be formalised^(1)^. Shortly after the discoveries of uranium and thorium, it was realised that they are not rare in the environment, uranium being several hundred times more abundant than gold and silver and thorium being about three times more abundant than uranium^(2)^. Both uranium and thorium can be found in many different minerals, 281 minerals are known today being associated with uranium and 25 associated with thorium^(3)^.

Among the decay products of uranium and thorium, radium was one of the elements that were widely recognized in the last century for their luminescence applications (e.g. radium watches). Unfortunately, radionuclides and their luminescence properties (e.g. uranium glassware) found their way into the health and beauty industries. Various claims were made that products containing radionuclides (e.g. radium water) could be beneficial for health. The manufacturers of these products clearly displayed the radioactive content and flaunted with how high the concentration was. Consequently, this also led to products being falsely but intentionally advertised as radioactive, to increase sales. Nowadays, these products are mostly referred to as ‘quack cures’^(4, 5)^.

Apart from products with radioactive substances, there are also various services that, for a fee, can provide a radiation exposure, usually in the form of radon inhalation. One facility claims the exposure of ~44 kBq m^−3^ of radon (presumably ^222^Rn) for 60 min (one session), can have therapeutic effects (radon activating a messenger in the body that inhibits inflammation and encourages healing). They also claim to have calculated the radiation dose from 10 to 12 sessions to be 1.8–2.2 mSv^(6)^. It can be mentioned that in a report by the International Atomic Energy Agency (IAEA), it is recommended to have an action plan for dwellings where ^222^Rn exceeds 300 Bq m^−3^, which would correspond to an annual effective dose of the order of 10 mSv^(7)^.

Thus, the manufacturing and provision of ‘quack cures’ has never ceased since there is still a demand from the public, despite the increased awareness we have today of the dangers of improper use of radioactivity. One of the differences regarding the advertisement (for selling) of these products compared to the last century is that words such as radium or radioactivity are to be avoided since this would alert authorities and sellers might face legal consequences. Furthermore, the increasingly growing online market facilitates for manufacturers to sell their products on a global scale. Since courier companies have limited to no possibility to measure packages whether they contain radioactive substances, little can be done to prevent illegal import and export of these products.

Recent social media feed has brought to our attention several commercially available products that are claimed to improve health and wellness through their mineral, ‘frequency’ or ‘energy’ contents and that are also radioactive^(8, 9)^. The measurements reported in social media seem to have been performed with relatively simple Geiger–Müller tubes and NaI detectors, and the activity concentration is, as far as we know, still unknown. Therefore, the aim of this study was to analyse some items available on the market, by gamma and alpha spectrometry, to identify and quantify potential radionuclide content and estimate the effective dose contribution from use of the items.

## Material and methods

**Figure 1:**
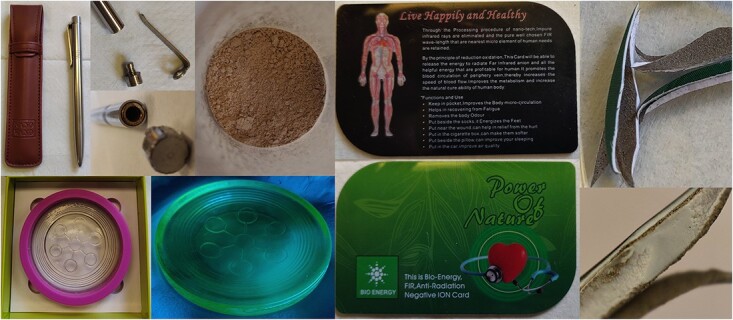
Pictures showing the three products that were included in this study: (a) stainless-steel pen containing a fine powder, (b) glass disc with a rubber ring also shown exposed to UV light and (c) plastic card (front and back) with two coatings of powder on the inside that can be seen in the cross section.

An online store was browsed for products that are claimed to benefit health in some aspect and with the keywords nano and scalar and that have been identified by previous investigators to contain radioactivity^(8, 9)^. Three products matched the previous description and can be seen in Figure 1.

### Stainless-steel pen

The stainless-steel pen was in one piece, weighing 48 g and had a metal plug tightly fitted in the top (with no rubber sealing) (Figure 1a). The metal plug was carefully removed with a chisel and a hammer, and the inside of the pen contained 7.214 g of densely packed beige-grey powder, which was further analysed. Some recommendations on the usage of the pen, according to the product description, included stirring a glass of drinking water with the pen and claim that consumption of such water should ‘rejuvenate’ the molecular structure of the body.

### Glass disc

The glass disc had a diameter of 89 mm and thickness of 10 mm, weighed 185 g and had a concave and a convex side and an outer rubber ring (Figure 1b). The disc was exposed to ultraviolet (UV) light (Philips TL 40 W/12) for visual detection of any fluorescent content, as seen in Figure 1b. The product description recommended a direct contact of drinking water with the glass disc, claiming that this procedure would ‘energise’ the water. Thus, both the rubber ring and the glass disc were analysed.

### Plastic card

The plastic card had the dimensions 85 × 54 × 1.5 mm^3^ and weighed 9.8 g (Figure 1c). According to the product description, it was recommended to place the card in a pocket and claimed that this procedure would remove body odour and improve body circulation. The card was cut into small pieces to analyse the content, and the layers quickly separated once it was cut (seemingly not held together by glue), revealing two coatings of a beige-grey powder on the inside, as seen in Figure 1c.

### Water samples

In order to study any surface contamination on these products, 291 g of distilled water was used to rinse the glass disc, rubber ring and plastic card, and the rinsing water was analysed as one sample. Furthermore, following the product description of the stainless-steel pen, the pen was submerged into a glass beaker with 515 g of distilled water for an hour with occasional stirring.

### Sample composition

In order to determine the activity concentration by gamma spectrometry, the efficiency calibration of the detector was adjusted for the composition and density of the measured sample. Therefore, some assumptions about the composition of the samples had to be made (Table 1). The composition of the glass disc was derived based on data by Mueller *et al.*^(10)^, and if any radionuclides were present in the glass, they were added to the composition by converting the activity concentration (obtained by alpha spectrometry) to mass concentration. The latter was calculated from the natural abundance of the radionuclides (1 ppm of U and Th corresponds to 12.4 and 4.06 Bq kg^−1^ of ^238^U and ^232^Th, respectively). The composition of the powder in the card and in the pen was assumed to be similar, based on a similarity in colour and texture. However, since the card was mainly composed of a plastic material, which was assumed to be polyvinyl chloride (PVC), the composition was scaled based on the amount of plastic compared to the powder coating. The scaling factor was obtained by measuring the width of the different layers in the enlarged picture (×10 magnification) in Figure 1c, where the two powder coatings were estimated to be 0.16 mm in total and the plastic (two outer layers and the middle piece) was 1.34 mm (a total of 1.5 mm, which was the thickness of the card). The powder was assumed to be some form of by-product obtained from mineral processing of rare earth metals, presumably monazite sand, which is known to contain up to 25% of Th^(11)^. Thus, the composition of monazite sand was taken to be the average of that given by Catlos and Miller^(12)^ and Nagy and Draganits^(13)^.

**Table 1:** The assumed composition for the samples. Data for the powder were taken from Catlos and Miller ^(13)^ and Nagy and Draganits^(12, 13)^; data for the glass disc were based on Mueller *et al*.^(10)^. The concentration of Th was updated following the results obtained in this study by alpha spectrometry.

Powder inside the pen		Plastic card		Glass disc	
P_2_O_5_	27.6%	C_2_H_3_Cl (PVC)	88.4%	SiO_2_	54.0%
Ce_2_O_3_	25.2%	P_2_O_5_	3.1%	Th	0.4%
Th	15.0%	Ce_2_O_3_	2.9%	CaO	17.2%
La_2_O_3_	13.5%	Th	1.7%	MgO	4.5%
Nd_2_O_3_	9.8%	La_2_O_3_	1.5%	Al_2_O_3_	14.0%
Pr_2_O_3_	2.5%	Nd_2_O_3_	1.1%		
Y_2_O_3_	1.9%	Y_2_O_3_	0.2%		
CaO	1.4%	CaO	0.2%		
Sm_2_O_3_	1.4%	Sm_2_O_3_	0.2%		
Gd_2_O_3_	1.1%	Gd_2_O_3_	0.1%		

### Analytical techniques

Alpha and gamma spectrometry was used to study the activity concentration of radionuclides in the naturally occurring ^232^Th and ^238^U decay series and any anthropogenic radionuclide that could be detected. All samples were prepared and measured during July 2021, and results are given at the time of measurement. All samples were measured until the counting uncertainty was less than 1% or for a maximum of 2 d. The uncertainty in the activity concentration was calculated as the combined uncertainty of the counting statistics, uncertainties in the measured weights and from the calibration of the detectors.

#### Gamma spectrometry

The samples were placed in cylindrical 5-ml containers and measured with a coaxial P-type high-purity germanium detector with an energy resolution of 1.65 keV at 1.33 MeV and with a relative efficiency of 52% at 1.33 MeV (GEM 50P4, ORTEC, Ametek, Inc., Oak Ridge, TN, USA). Three containers were filled with the following samples (without any prior drying): the powder from the pen, the glass disc, which was milled into a fine powder, and the plastic card. For the latter, the card was cut into circular pieces fitting the 5-ml container, since the layers in the plastic card were easily separated after cutting it into small pieces (leaving behind pieces with varying amounts of powder), in order to maintain the integrity of the card. The 5-ml containers were filled with the crushed glass and half-filled with the powder from the pen and with the circular pieces of the plastic card, respectively. The containers were stored for ~3 weeks to allow the in-growth of ^222^Rn. However, the containers were not sealed in Rn-tight bags, due to the very small container size that would hinder a reproducible geometry. The freely available software EFFTRAN was used to produce the necessary correction of the efficiency calibration for the calculation of activity concentration^(14)^. This included efficiency curves, corrections for coincidences and self-absorption, based on the assumed compositions (Table 1).

#### Alpha spectrometry

The following six samples were analysed: the powder from the pen, the finely crushed glass, the rubber ring cut into thin pieces, the plastic card cut into small pieces, the water sample in which the pen was submerged into and the water used for rinsing the other products. Appropriate sample weights were estimated from the activity concentrations obtained by gamma spectrometry, in order to maintain a good energy resolution. The samples were then digested by microwave-assisted digestion (Ethos Easy, Milestone S.r.l.) with appropriate acids (Fisher Chemical™, Thermo Fisher Scientific, MA, USA) to fully dissolve the sample. Since the composition of the samples were unknown, several trials were made with different volumes of acids and molarities until no visible residue was seen in the digested aliquot. For the powder and glass, hydrofluoric acid and *aqua regia* were used, with a second digestion adding boric acid for complexation of fluoride and hydrogen peroxide for additional digestion of remaining organics. A similar procedure was adapted for the plastic card, however, with the addition of sulfuric acid. For the rubber ring, the digestion was based on ISO 9028^(15)^ using sulfuric acid and *aqua regia*.

The separation of U, Th, Ra and Po was achieved with Tri-n-butyl phosphate (TBP), Ra and Po were eluted with 6 M HNO_3_, Th with 4 M HCl and U with distilled water (in that order). If any other actinides such as Am, Cm or Pu were present in the samples, they would be identified (to a limited degree) in the U and Th spectra. Most of the Am and Cm would, in that case, be found in the Ra and Po fraction since only small amounts of Am and Cm are extracted by TBP. The activity and chemical yield were determined by the addition of tracers prior to the digestion: ^232^U, ^229^Th, ^225^Ra and ^209^Po for U, Th, Ra and Po isotopes, respectively. The samples were measured by ion implanted silicon-charged particle detectors (Alpha Ensemble® ORTEC) where the discs for U and Th isotopes were prepared by micro coprecipitation with CeF_4_, ^226^Ra with BaSO_4_ and Po isotopes by spontaneous deposition on copper discs.

### Assessment of radiation exposure

The committed effective dose was calculated if any activity concentration was detected in the water samples, using dose coefficients for ingestion, given by ICRP 119^(16)^. The effective dose was also calculated for the workers exposed to potential radioactive particles (through inhalation). For the latter, since no details were known regarding particle size and air concentration, data on working conditions were taken from ICRP 66^(17)^ and based on the study on workers exposed to radioactive particles in phosphate industry by Kim *et al*.^(18)^. It was then assumed that the air breathed during a working day is 9.6 m^3^^(17)^ and that the size of the aerosols was 5 μm with a concentration of 1 μg m^−3^ in the air^(18)^. In this calculation, a moderate rate of absorption (absorption type M) was generally assumed, but if no value for moderate absorption was available, data for fast absorption (type F) were used instead.

Furthermore, an assessment of the external radiation exposure from the products was performed by the WISE Uranium Project Calculator^(19)^. The pen, card and glass disc were assumed to be point sources for simplicity. Since the pen had a pocket clip, both the pen and the card were assumed to be worn close to the body at a distance of 5 cm, while the glass disc was assumed to be at 1 m distance. The card and pen were assumed to have a shielding of half of their thickness, 0.75-mm plastic and 1.2-mm shielding of stainless steel (SAE 304, with 69.5% Fe, 19% Cr, 9.5% Ni and 2% Mn). The glass disc was also assumed to have a shielding of half its thickness, 5-mm glass, although the activity is most likely homogeneously distributed within the glass. These choices were selected for simplifying the calculations and due to limitations in the used calculator. The receptor material was assumed to be tissue according to the composition of ICRU 44^(20)^.

## Results

### Radionuclide concentration in the samples

**Figure 2:**
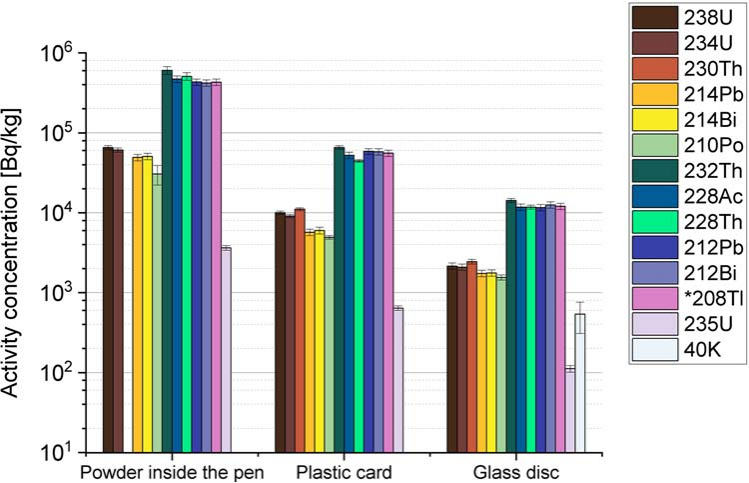
Activity concentrations in becquerels per kilogram of the powder inside the pen, the plastic card and the glass disc, obtained by alpha and gamma spectrometry. The error bars represent the combined uncertainty (*k* = 1) of the counting statistics, the measured weight and the calibration of the detector. The activity concentration for *208Tl was scaled by the factor 0.3594 to take into account the branching ratio in the decay of 212Bi. The activity concentration of 230Th could not be determined in the powder due to difficulties in assessing the peak area.

**Figure 3:**
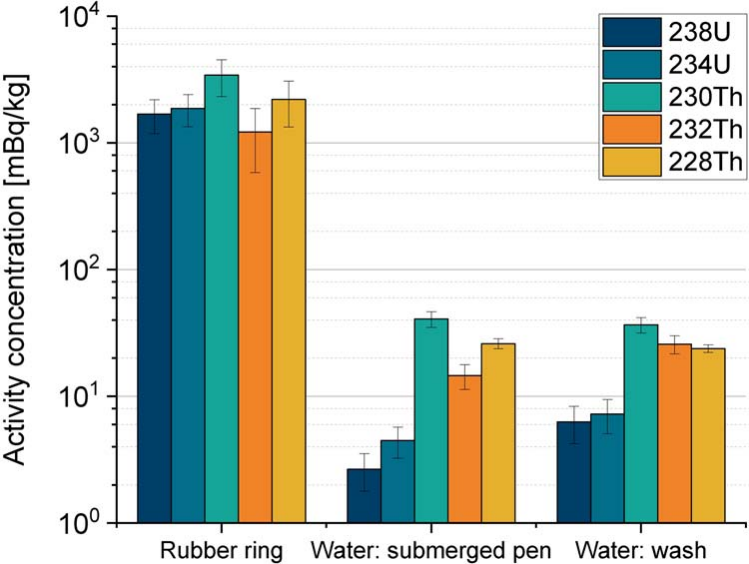
Activity concentration in megabecquerels per kilogram of the rubber ring and the two water samples (stainless-steel pen submerged into one of the beakers and the water used to rinse the plastic card, rubber ring and glass disc). The results were obtained by alpha spectrometry, and 226Ra and 210Po values were below the detection limit (~2 mBq kg^−1^). The error bars represent the combined uncertainty (*k* = 1) of the counting statistics, the measured weight and the calibration of the detector.

No anthropogenic radionuclides were found in the samples, only the naturally occurring radionuclides that are shown in Figures 2 and 3. Figure 2 shows the results obtained by both alpha and gamma spectrometry, while Figure 3 includes only results from alpha spectrometry. All uncertainties are given as expanded uncertainty with a coverage factor of 1 (*k* = 1).

For alpha spectrometry of U and Th isotopes, the detection limit ranged from 1 to 36 Bq kg^−1^ for the plastic card, glass disc and rubber ring. For ^210^Po, the detection limit ranged from 50 to 130 Bq kg^−1^ for the plastic card, glass disc and rubber ring. For the two water samples, the detection limits for U, Th, Ra and Po were ~2 mBq kg^−1^. For the powder inside the pen, the detection limits were higher due to the much lower mass used and the low chemical yield: ~20 Bq kg^−1^ for U isotopes, 500 Bq kg^−1^ for ^210^Po and 12 600 Bq kg^−1^ for Th isotopes. For gamma spectrometry, the detection limit for the radionuclides of interest was ~200–3000 Bq kg^−1^ (200 000 Bq kg^−1^ for ^210^Pb) for the powder inside the pen, 50–300 Bq kg^−1^ for the plastic card and 3–50 Bq kg^−1^ for the glass disc. ^40^K was only detected in the glass disc, and the detection limit for this radionuclide was 800 and 150 Bq kg^−1^ in the powder and the plastic card, respectively. The detection limits are given with a 95% confidence level.

The results in Figure 2 show that all products contained detectable amounts of the naturally occurring radionuclides found in the ^238^U and ^232^Th decay series. The radionuclide with the highest activity concentration of 0.61 MBq kg^−1^ was ^232^Th, which was found in the powder inside the pen. This corresponds to ~15% Th by weight (1 ppm corresponds to ~4.07 Bq kg^−1^). The radionuclides in the ^238^U decay series were about one order of magnitude lower than those in the ^232^Th decay series. The peak area of ^230^Th could not be accurately determined in the powder of the pen due to the relatively high activity concentration of ^229^Th (positioned close to ^230^Th) and the low chemical yield; thus, the activity concentration of ^230^Th is not shown in Figure 2. The activity concentration of ^234,238^U and ^228,230,232^Th was ~1 Bq kg^−1^ in the rubber ring and ~3–40 mBq kg^−1^ in the two water samples. Table 2 shows the calculated ratios for the alpha and gamma emitters in the ^232^Th and ^238^U decay series in the powder inside the pen, plastic card and glass disc.

**Table 2:** Ratios of the activity concentration of radionuclides in the ^238^U and ^232^Th decay series in the powder inside the pen, plastic card and glass disc. The results were obtained by alpha and gamma spectrometry.

	Ratios in the ^238^U chain						
	^238^U/^234^U	^234^U/^230^Th	^230^Th/^214^Bi	^214^Bi/^210^Po	^238^U/^214^Bi	^238^U/^210^Po	
Powder	1.1 ± 0.1			1.7 ± 0.5	1.2 ± 0.1	2.1 ± 0.6	
Card	1.1 ± 0.1	0.8 ± 0.1	1.9 ± 0.2	1.2 ± 0.1	1.5 ± 0.2	2.0 ± 0.1	
Glass	1.0 ± 0.1	0.9 ± 0.1	1.4 ± 0.2	1.1 ± 0.2	1.2 ± 0.2	1.4 ± 0.2	
Ratios in the ^232^Th chain			Ratio indicative of the origin		Ratios for internal validation of the analytical techniques		
	^232^Th/^228^Ac	^228^Ac/^212^Bi		^232^Th/^238^U		^228^Th/^212^Bi	^212^Bi/^208^Tl
Powder	1.3 ± 0.2	1.1 ± 0.2	Powder	8.4 ± 1.1	Powder	1.2 ± 0.2	1.0 ± 0.1
Card	1.3 ± 0.1	0.9 ± 0.1	Card	6.8 ± 0.7	Card	0.8 ± 0.1	1.0 ± 0.1
Glass	1.2 ± 0.1	0.9 ± 0.1	Glass	6.1 ± 0.6	Glass	0.9 ± 0.1	1.0 ± 0.1

### Internal dose assessment

The total radioactivity that was rinsed off the glass disc, rubber ring and plastic card was (in megabecquerels) 7.5 for ^232^Th, 6.9 for ^228^Th, 1.8 for ^238^U, 2.1 for ^234^U and 11 for ^230^Th. Similarly, in the total activity that was rinsed off (or leached) from the pen being submerged into, and stirring, the water was (in megabecquerels) 7.5 for ^232^Th, 13 for ^228^Th, 1.4 for ^238^U, 2.3 for ^234^U and 21 for ^230^Th. If these two water samples are ingested, the committed effective dose to an adult would be 12 nSv, considering only the detected radionuclides.

For the workers’ exposure, inhaled aerosols were assumed to have the same activity concentration as the analysed powder in the pen. Furthermore, the decay products in the ^232^Th and ^238^U were assumed to be in equilibrium since the measurement data indicated that this was indeed the case; see Figure 2. Thus, their activity was also included in the calculation if coefficients for them were available. From the dose coefficients in ICRP 119^(16)^, the effective dose to workers from 8 h of exposure was calculated to 0.39 mSv. The dominating radionuclide contributing to the committed effective dose was ^232^Th: an assumed inhalation of 9.6 μg of the powder (during a working day) corresponds to ~5.8 Bq, and with a dose coefficient of 0.029 mSv Bq^−1^, this corresponds to 43% of the calculated effective dose. These estimations were made under the assumption that no protective equipment was used during the work, which is a worst-case scenario.

### External dose assessment

For the external dose assessment (considering only the gamma radiation), all the decay products in the ^232^Th and ^238^U decay series were included, and the radionuclides that were not measured directly were either assumed to be in equilibrium with their progeny or decay product, which was measured. The calculated dose from wearing the pen was 440 nGy h^−1^ (34 nGy h^−1^ from ^238^U series and 400 nGy h^−1^ from ^232^Th series), and from wearing the card, 110 nGy h^−1^ (9 nGy h^−1^ from ^238^U series and 100 nGy h^−1^ from ^232^Th series), and from the glass disc, 8.4 nGy h^−1^ (0.1 nGy h^−1^ from ^238^U series, 8.3 nGy h^−1^ from ^232^Th series and 0.04 from ^40^K). Assuming an exposure from all three products during 8 h per day would correspond to a total of 4.4 μGy per day and 1.6 mGy for a whole year.

## Discussion

### Sample preparation

The main challenge for working with these samples was choosing an appropriate sample size for alpha spectrometry, large enough for a low uncertainty in the weight but small enough to maintain a satisfactory energy resolution. Since the activity concentration was relatively high for the powder (both in the pen and in the plastic card), a sample size of 1 mg had to be used for the powder. The precision scale used had an accuracy of ±0.1 mg, and for 1 mg of sample, the uncertainty was a minimum 10% (for a coverage factor of 1). This can be seen in Table 2 where the uncertainties in the ratios for results obtained by alpha spectrometry are much higher compared to those obtained by gamma spectrometry only. Since large amounts of the ^229^Th had to be added for a good visualisation of the peaks in order to correctly determine ^232^Th and ^228^Th, the peak area determination of ^230^Th was not possible due to the interferences from the low-energy tail of ^229^Th.

Regarding the acids used for digestion, the addition of hydrofluoric acid (HF) to fully dissolve SiO_2_ compounds could not be used when analysing Th isotopes. Yields less than 1% were obtained, which could be due to the formation of Th-fluorides that could not be dissociated and thus Th could not be extracted into TBP. The addition of boric acid followed by another digestion did not increase the chemical yield for Th. Digestion methods including HF is commonly found in soil analysis, where large amounts of SiO_2_ compounds are present and need to be completely dissolved for analysis of the total concentration in the soil. A too low chemical yield of Th in soil analysis can be avoided due to relatively large amounts of SiO_2_ and rare earth metals present in the soil, which readily form fluoride complexes (competing with Th). However, for the powder in these products, the concentration of Th could be higher than that of SiO_2_ and rare earth metals so that the addition of HF would complex practically all Th. Thus, the digestion of the powder inside the pen and the plastic card were only performed with *aqua regia* when analysing Th isotopes. However, when analysing U (and Po) isotopes, HF was added to the digestion vessel since the addition of peroxide would oxidise U(IV) to U(VI) causing it to likely dissociate from any fluoride complexes and thus be extracted into TBP (as an HNO_3_-complex). The chemical yield for U isotopes in the powder (when using HF) was ~25%. Furthermore, since the activity concentration of U isotopes and ^210^Po were about one order of magnitude lower than that ^232^Th, a larger sample size (60 mg) could be used to reduce uncertainties in the sample weight and still maintain a satisfactory energy resolution.

The digestion of the glass disc had to use HF in order to dissolve the silicates. At first, a ratio of 6:1 of ml HF (48%) to gram of sample size were used, but small residues were visible after the digestion and the activity concentration of ^238^U was calculated to 1000 Bq kg^−1^. When the amount of HF was increased to a ratio of 8:1, no visible residue could be seen and the activity concentration of ^238^U was calculated to 2200 Bq kg^−1^, which is the value shown in Figure 2.

Assuming that the ratio of ^238^U/^234^U is in fact 1, the ratios in Table 2 (1.1 ± 0.1) show that the usage of TBP seems to have provided a satisfactory separation between Th and U isotopes, specifically ^232^Th and ^238^U. Both radionuclides have similar energies (4.0 and 4.2 MeV, respectively), and in a U-spectrum, it would be difficult to determine if any ^232^Th were present, since the tail of the ^238^U peak would cover the ^232^Th peak. Considering the activity concentration of ^232^Th in the powder, the separation is estimated to be better than 98.9%. This was calculated by assuming that the ratio of ^238^U/^234^U is 1 and that the ratio of 1.1 seen in Table 2 is due to contamination of ^232^Th in the ^238^U peak, which would correspond to a contamination of 1,1% (using the activity concentration of ^232^Th).

### Activity concentrations and ratios

The study of the activity concentration by both alpha and gamma spectrometry provided a good overview of the majority of the radionuclides in the ^232^Th and ^238^U decay series. In general, both ^232^Th and ^238^U decay series seems to be in equilibrium, although some minor disequilibrium can be seen in the ^238^U series. However, considering the uncertainties, the potential loss of Rn from the sample containers, and potential underestimation caused by the tracers added being in liquid form while the samples were in solid form (e.g., incomplete digestion of refractory particles), it is assumed that there was an equilibrium in the two decay chains. Furthermore, since HF acid was not used when analysing Th (except for the glass), the radionuclides had to be analysed separately, using two different aliquots. Moreover, small sample sizes had to be used when analysing Th due to the relatively high activity concentration that could have led to lack of reproducibility when comparing U and Po with Th.

The alternative hypothesis would be that there is a disequilibrium in the ^238^U chain, which would indicate that the material used in these products has been exposed to some (chemical) processing. If that were the case, then it can be assumed that the elements U, Th, Ra and Pb and their different chemical behaviour would likely cause a much more apparent disequilibrium than that seen in Table 2. The largest disequilibrium within the same chain was seen for the ratio ^238^U/^210^Po with a value of 2 for the powder and the card. This could indicate that the powder used in both products has been exposed to high temperatures (e.g. ashing) that could release the readily volatile element Po. Since a disequilibrium is not seen in the glass, it could indicate that the glass disc was manufactured much earlier than the powder in the pen. This disequilibrium could be confirmed by analysing the products again after ~2 y, when ^210^Po would be in equilibrium with ^210^Pb (a full ingrowth). If the ratio then is still not 1, it would suggest a disequilibrium for ^210^Pb. The analysis of ^210^Pb by gamma spectrometry could provide insight on possible disequilibrium, however, due to the high activity concentration of several gamma emitters in the sample, the detection limit of ^210^Pb was 200 000 Bq kg^−1^ (a factor three times higher than the assumed activity concentration of ^210^Pb in the sample) for the detector used (P-type coaxial germanium detector).

Regarding gamma spectrometry, the activity concentration of ^228^Ac in the powder was sufficiently high so that its gamma rays with intensities as low as 0.1% were readily visible. Since ^228^Ac has more than 200 registered gamma rays, the gamma spectrum was quite complex (also including summation peaks) and only radionuclides with several visible gamma rays were included in the calculation so that several peaks could be verified with the calculated activity concentration. This excluded analysis of e.g., ^234m^Pa (peak at 1001 keV and intensity 0.85%), which would have been a valuable peak to verify the activity concentration of ^238^U. Around this energy, ^228^Ac also has a gamma ray (1000.7 keV) with an intensity of 0.0054%, and considering the activity concentration of ^228^Ac, this contributes to ~6% of the peak area (excluding corrections for coincidences). However, ^40^K (1461 keV) in the glass disc was analysed despite the fact that only one gamma ray is available. ^228^Ac also has a peak at this energy (1459 keV with intensity 0.87%), which was manually subtracted from the peak area and corresponded to 48% (hence the relatively high uncertainty for ^40^K).

Concerning the ratio ^228^Th/^212^Bi of 1, as seen in Table 2, this indicates a good agreement between the results obtained by alpha and gamma spectrometry. Furthermore, the ratio ^212^Bi/^208^Tl indicates that the calculations (efficiency curve and coincidence corrections) by gamma spectrometry were performed correctly. Another interesting ratio is ^232^Th/^238^U, where all three products seem to have a similar ratio considering the uncertainties and errors mentioned earlier. It is unlikely that this ratio would be equal for these products if they were not of the same origin. Hence, the conclusion is that the radioactive source found in these products is the same and has been incorporated in various amounts in the pen and the plastic card and melted into the glass.

### Dose assessment

From the dose assessment, the major concern is primarily for the workers coming into contact with the powder. Furthermore, since a few megabecquerels of several alpha emitters were detected in the water samples that the products came into contact with (indicating a surface contamination), the concern is also for the workers packaging these products who may not be aware of surface contamination.

For the consumers of these products, the main concern is the stainless-steel pen and the plastic card where the powder from both these products could potentially be released after some wear and tear. Especially, the pen that had the highest activity concentrations intended to be submerged into a glass of water prior to drinking and with no rubber sealing (which is used for waterproofing). The powder was kept inside the pen by a metal plug only, and concerns are raised for the potential release of the fine powder from the metal–metal sealing.

The committed effective dose from drinking the two water samples was calculated to 12 nSv, and even if all radionuclides in the decay series were included, the dose could still be considered negligible compared to the average annual effective dose of 2.4 mSv received from natural sources^(21)^. However, this could be considered as a one-time exposure, where the dose could be scaled with the number of times these products are used with drinking water. Moreover, it is not known whether this activity will be released in the same amount at each use, if an increased amount would be released with time due to wear and tear or if the amount would decrease after removal of the initial surface contamination.

## Conclusion

This study confirms that there exist products that are readily available for purchase from the online market, and are claimed to improve health and wellness, and contain relatively high concentrations of naturally occurring radionuclides. The product descriptions do not mention the radionuclide content, and concerns are raised for the consumers and workers exposed to these products with no knowledge of the radioactivity levels. Moreover, the products were contaminated on the surface, and it is unknown how well the integrity of the products holds with time.

The powder in the stainless-steel pen contained the highest activity concentration of ^232^Th and ^238^U, 0.61 and 66 kBq kg^−1^, respectively, and weighed 7.214 g. This corresponds to a content of ~4.4 and 0.48 kBq of ^232^Th and ^238^U, respectively. The current listing price for the pen is ~9.5 USD, which would correspond to a selling price of ^232^Th and ^238^U of 0.46 and 0.05 kBq per USD, respectively and is readily available for anyone to purchase. Considering the production of so-called dirty bombs (explosives containing radionuclides), this aspect is perhaps the biggest concern worldwide.

## Data Availability

All data generated and analysed during this study are included in this published article.

## References

[1] Malley, M. C. Radioactivity a history of a mysterious science. (New York: Oxford University Press) (2011).

[2] Krebs, R. E. The History and Use of Our Earth's Chemical Elements: A Reference Guide (Westport, Connecticut: Greenwood Press)(2006).

[3] Mindat . The world’s leading authority on minerals and their localities, deposits, and mines worldwide. Online database, mindat.org. (06 November 2019).

[4] Blaufox, M. D. Radioactive artifacts: historical sources of modern radium contamination. J Med Imaging Radiat Sci 50(4), S3–s17 (2019). 10.1016/j.jmir.2019.11.004 31862163

[5] Horowitz, S., Rogers, P. and Auer, C. Bulletins. Bull. At. Sci. 58(3), 6–12 (2002).

[6] Gastein Healing Gallery Therapy & Health Center . www.gasteiner-heilstollen.com/en Accessed June 2022.

[7] International Atomic Energy Agency . Radiation Protection and Safety of Radiation Sources: International Basic Safety Standards GSR Part 3. (Vienna: IAEA) (2014).

[8] The Thought Emporium . Content creator on social media (2021) Available from: https://www.thethoughtemporium.com/.

[9] Big Clive . Content creator on social media. (2021). Available from: http://www.bigclive.com/.

[10] Mueller, J. R., Boehm, M. W. and Drummond, C. Direction of CRT waste glass processing: electronics recycling industry communication. Waste Manag. 32(8), 1560–1565 (2012). 22465397 10.1016/j.wasman.2012.03.004

[11] Hoshino, M., Sanematsu, K. and Watanabe, Y. *Chapter 279 - REE Mineralogy and Resources*. In: Handbook on the Physics and Chemistry of Rare Earths. Jean-Claude, B. and Vitalij, K. P., Eds. ( Elsevier) pp. 129–291 (2016).

[12] Catlos, E. J. and Miller, N. R. Speculations linking monazite compositions to origin: Llallagua tin ore deposit (Bolivia). Resources 6(3) (2017). 10.3390/resources6030036.

[13] Nagy, G. and Draganits, E. Occurrence and mineral-chemistry of monazite and rhabdophane in the lower and? Middle Austroalpine tectonic units of the southern Sopron Hills (Austria). Mitteilungen der Gesellschaft der Geologie- und Bergbaustudenten in Österreich 42, 21–36 (1999).

[14] Vidmar, T. EFFTRAN: Efficiency transfer and coincidence summing corrections for environmental gamma-ray spectrometry. Available from: http://efftran.com/. 10.1016/s0969-8043(99)00246-8 10724442

[15] International Organization for Standardization (ISO) . Rubber – Dissolution by acid digestion (9028), 1st edition 1989-08-01 edn.

[16] Eckerman, K., Harrison, J., Menzel, H. G. and Clement, C. H. ICRP publication 119: compendium of dose coefficients based on ICRP publication 60. Ann. ICRP 42(4), 1–e130 (2013). 10.1016/j.icrp.2012.06.038 23025851

[17] International Commission on Radiological Protection . Human respiratory tract model for radiological protection. A report of a task group of the ICRP. Ann. ICRP 24(4), vii–482 (1994). 7726471

[18] Kim, K. P., Wu, C. Y., Birky, B. K. and Bolch, W. E. Influence of particle size distribution on inhalation doses to workers in the Florida phosphate industry. Health Phys. 91, 58–67 (2006). 16775481 10.1097/01.HP.0000200261.96014.6c

[19] WISE Uranium Project . External Radiation Dose Calculator (Virtual Geiger Counter), last updated 30 April 2021. Available from: http://www.wise-uranium.org/rdcx.html.

[20] White, D. R., Booz, J., Griffith, R. V., Spokas, J. J. and Wilson, I. J. Report 44. Journal of the International Commission on Radiation Units and Measurements os23(1), NP (1989).

[21] UNSCEAR . *Sources and effects of ionizing radiation*. In: Report to the General Assembly with Scientific Annexes. Vol. I. ISBN 978-92-1-142274-0. (2008).

